# Lydston’s Views of the Race Problem

**Published:** 1909-12

**Authors:** G. Frank Lydston

**Affiliations:** Chicago


### Correspondence.

### Lydston's Views of the Race Problem.

*Editor Texas Medical Journal:*

Garbled interviews, misleading sensational headlines and inaccurate quotations from my lectures and writings on sociologic topics by the secular press have frequently misrepresented my views of various subjects. None has been so willfully misrepresented as my attitude toward the race problem. I have repeatedly been made to say that I advocated miscegenation. This is utterly false. Numerous journals and magazines already have quoted my views on certain phases of the race problem, the tone of which was alone sufficient to refute this absurd deduction from my work. I have discussed the subject in my "Diseases of Society and Degeneracy" in a manner which should set at rest all question as to my attitude. Briefly, my views are as follows:

The race problem did not begin with the landing of the first slave on American soil, nor with the liberation of the slaves. It began with the outrage upon the South of attempting to place the ignorant freedman on a political equality with the whites. In this attempt tradition, custom and local conditions were set at naught. The fourteenth and fifteenth amendments to the Constitution are tantamount to an agreement on the part of the United States to deliver something which it never has been, and may never be, able to deliver, either in the North or South, least of all in the South.

The Southerner, the man on the social and political firing line, was confronted with the alternatives of social and political submergence through acquiescence with the provision of the Constitution, and defending his vested rights by evading the constitutional big stick as best he might. He chose the latter alternative, as any race of white men in a similar position would have done, for, all theories aside, this was and is a white man's country.

In the light of experience, the constitutional amendments in question are a farce and a menace. We can evade the issue, but—after us the deluge—our posterity will pay dearly for the political knavery, impracticability and evasion of issues of their ancestors. A social fire is no less a fire because it is smothered by "bunk" and evasion.

The North has vituperated and berated the South for its attitude toward the negro, but it is obvious that the regulation of one's household by a neighbor is rarely a success. The family may not always be right, but it knows far better than the neighbors the best method of meeting the exigencies of its daily life. The man on the firing line best knows the most effective method of attack and defense.

Legislation against miscegenation is illogical and ineffective because it bars legitimate relations of whites and blacks and does not penalize illegitimate relations. Legislation should go to its logical ultimate; otherwise it puts a premium on illegitimate relations. The job should be done thoroughly if undertaken at all. To what extent may race amalgamation eventually occur in this country? No man can say. When we consider that from 20 to 25 per cent of avowed negroes in America have white blood in their veins, to say nothing of those who have so much white blood that they pass for whites, it does not require much profundity of thought to suggest what may happen. Legislation against animality, against the primitive instinct of sexual selection, has failed. The first step toward amalgamation has been taken. The mingling of bloods has gone on, is going on and will continue, possibly at a geometric rate of increase. The black stream has always contaminated the white stream at its borders. These borders have become yellow and yellow-white, and are encroaching more and more upon the central stream. If the white stream and the black and yellow stream remain segregated, there will always be a race problem. If they amalgamate the race problem will disappear, but race degeneracy and perhaps extinction for the amalgamated breed will replace it. Think it over dispassionately and answer me: Can we always stave off the issue? Will not our children and our children's children pay the price of the lunacy of their ancestors? Are we not the traditional ostrich with his head in the sand? In short, do we not stand between the Scylla of the Race Problem and the Charybdis of Amalgamation? One or the other will eventually prevail and bring disaster. The only alternative would be sterilization of the entire negro race, and he would be bold indeed who would dare to advocate this. Humanity at large would never tolerate such a thing.

The negro is becoming educated—education brings ambition.

He is becoming well-to-do—wealth brings power. He is becoming a political factor—this brings more power. Supposing he should demand the fulfillment to the letter of the provisions of the fourteenth and fifteenth amendments. Would he get what he demanded? Hardly. The North could not "deliver the goods," and the South would not. Alas, poor old Government. But this never can happen in our day—therefore we are comfortable. Again,—"After us the deluge." But it will one day happen unless amalgamation of races occurs, in which event—well, some more "after us the deluge." It is not pleasant to contemplate the prospect of America being populated by an unhappy mixture of races showing physical and mental deterioration as its dominant characteristic, with racial extinction looming up as a dark background to the dark picture. Let it be remarked here and now that the North, not the South, will be the first to show the evil results. Social and political segregation in the South are less easily surmountable barriers than any which would have to be climbed in the admixture of bloods in the North. In some ways the North is more tolerant of the negro than is the South, but the intolerance is not so practical so far as segregation and white social self-defense is concerned.

History and sociologic research alike bear me out in my views of the race problem. Only the ignorant or viciously disposed could construe my views as an endorsement of miscegenation.

Frankly, I am not one of those pink-eyed optimists who believe that the race problem, if it endures at all as such, will take care of itself. I am very pessimistic, and can see nothing but breakers ahead—issues which must one day be met squarely, fairly and courageously. Whether enormous expenditures of money will be flanked by blood or tears, or by both, I know not, but I like comfort and peace of mind and am just selfish enough to be glad that I shall not be here to estimate the exact price posterity will pay for the sins and follies of its fathers.

G. Frank Lydston.

Chicago, November, 1909.

